# Factors regulating cellulolytic gene expression in filamentous fungi: an overview

**DOI:** 10.1186/s12934-022-01764-x

**Published:** 2022-03-22

**Authors:** Anu Jose Mattam, Yogesh Babasaheb Chaudhari, Harshad Ravindra Velankar

**Affiliations:** Hindustan Petroleum Green R and D Centre (HPGRDC), KIADB Industrial Area, Tarabanahalli, Devanagundi, Hoskote, Bangalore, 560067 India

**Keywords:** Cellulase expression, Transcription factors, Regulation, Transporters, Catabolite repression

## Abstract

The growing demand for biofuels such as bioethanol has led to the need for identifying alternative feedstock instead of conventional substrates like molasses, etc. Lignocellulosic biomass is a relatively inexpensive feedstock that is available in abundance, however, its conversion to bioethanol involves a multistep process with different unit operations such as size reduction, pretreatment, saccharification, fermentation, distillation, etc. The saccharification or enzymatic hydrolysis of cellulose to glucose involves a complex family of enzymes called cellulases that are usually fungal in origin. Cellulose hydrolysis requires the synergistic action of several classes of enzymes, and achieving the optimum secretion of these simultaneously remains a challenge. The expression of fungal cellulases is controlled by an intricate network of transcription factors and sugar transporters. Several genetic engineering efforts have been undertaken to modulate the expression of cellulolytic genes, as well as their regulators. This review, therefore, focuses on the molecular mechanism of action of these transcription factors and their effect on the expression of cellulases and hemicellulases.

## Introduction

The gradual depletion of conventional fossil fuels and increasing awareness about the effects of greenhouse gas emissions has led to global interest in the development of renewable energy, particularly biofuels such as ethanol. Ethanol can be blended with gasoline up to 20% (v/v) and used in conventional motor engines, without any effect on the engine performance [1], thereby leading to reduced consumption of fossil fuels and lower emissions. Biofuels can be categorized into 1st, 2nd, 3rd, and 4th generation biofuels based on the feedstock used for production. The 1st generation biofuels are produced by the fermentation of sugar or starchy substrates [2] and these processes have already been commercially established. However, considering the huge demand for ethanol, it has become necessary to expand the scope of fermentable substrates beyond the conventional ones (e.g. cane molasses, starch, etc.) to lignocellulosic biomass, which is available in abundance. Depending upon their geographical locations, lignocellulosic feedstock suitable for biofuel production can vary from grasses (miscanthus and switchgrass), crop residues (rice straw, wheat straw, cotton waste, etc.), weeds (such as *Eicchornia crassipes*, *Lantana* *camara*, *Prosopis uliflora, Saccharum spontaneum, Crofton* and *Chromolaena odorata*) to woody biomass (*Pinus, Populus, Pseudotsuga*, Aspen, etc.) [3–7].

Lignocellulosic biomass consists of cellulose (35–50%) surrounded by a highly cross-linked network of hemicellulose (20–35%) and lignin (10–15%) [8]. The lignin–hemicellulose network has to be disrupted by thermochemical pretreatment to release cellulose—a complex carbohydrate, containing glucose units linked to each other by covalent β-glycosidic bonds. In addition, the extensive network of intra- and inter-molecular hydrogen bonds in cellulose leads to increased cellulose crystallinity and reduced amenability to hydrolysis [9]. Acids are effective catalysts that can overcome the recalcitrance of cellulose under certain conditions, however, their use for biomass hydrolysis usually results in the formation of sugar dehydration products such as furfural, 5-hydroxymethylfurfural, and other furans which are inhibitory to microbial growth and fermentation [10].

The enzymatic hydrolysis of cellulose into glucose by the action of cellulases occurs under mild conditions and is both environment-friendly and sustainable due to the possibilities presented by enzyme reuse/recycling techniques. Cellulolytic enzymes or cellulases are a group of hydrolytic enzymes consisting of endoglucanases (EC 3.2.1.4), cellobiohydrolases (EC 3.2.1.91), and β-glucosidases (EC 3.2.1.21). The endoglucanases catalyze the cleavage of internal β-glycosidic bonds of the cellulose chain to generate cello-oligosaccharides while the cellobiohydrolases act on both, the reducing and non-reducing ends of cellulose or cello- oligosaccharides to generate cellobiose units (2 glucose molecules linked by a β 1 → 4 linkage) [11, 12]. The β-glucosidases hydrolyze cellobiose and cellodextrin units to release monomeric glucose units [13–15]. Several accessory proteins such as swollenins, lytic polysaccharide monooxygenases, expansins, etc. also facilitate the hydrolysis of cellulose by enhancing the enzyme–substrate interaction in different ways [16].

Cellulases are produced by both, bacteria (*Pseudomonas fluorescens*, *Bacillus subtilis*, *Serratia marcescens*, etc.) [17] and filamentous fungi (e.g. soft-rot, white-rot and brown-rot fungi) [18]. Generally, fungi exhibit higher levels of protein expression and are therefore the preferred hosts for enzyme production [19]. Moreover, fungi also have an advanced machinery for carrying out the post-translational processing of proteins (e.g., glycosylation, protease cleavage, and disulfide bond formation, etc.), which are critical for imparting specific functions to these proteins [20]. Despite these advantages, a single cellulolytic fungal strain with the ability to express all classes of cellulases at sufficiently high concentrations is rare, and therefore, different components of cellulases are usually produced separately using different strains, and then blended. In comparison with yeast or bacterial fermentation, mycelial fermentations are usually difficult to control due to problems related to mass transfer [21, 22]. Specifically, cellulase expression by fungi such as *Trichoderma* requires the presence of insoluble substrates like cellulose or lignocellulosic biomass along with soluble medium components such as various salts during fermentation. Cellulose being sparingly soluble in water is not evenly dispersed in the fermentation medium resulting in non-uniform utilization by the fungi. Although the dispersion of cellulose can be enhanced by increasing the agitation rates, this is not preferred at larger scales due to the high energy requirement as well as the risk of damaging fungal mycelia in the process [23].

**Fig. 1:**
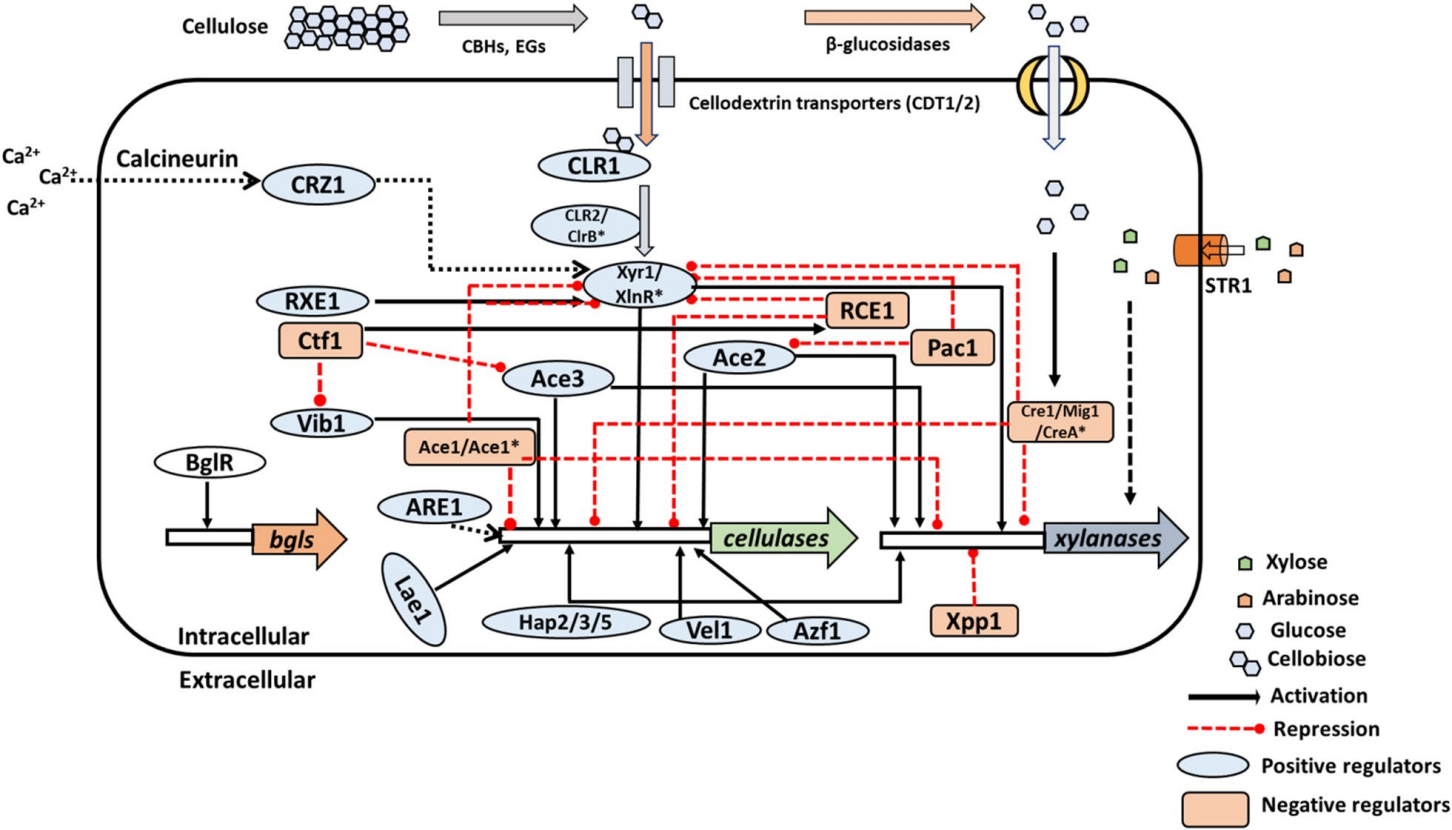
Transcriptional regulators and transporters involved in cellulase expression in *T. reesei* and *Penicillium* sp

As enhancing cellulase production by the optimization of process conditions for fungal fermentation, is limited by the use of insoluble substrates and the mycelial nature of the fungus, several genetic engineering efforts have also been attempted to improve cellulase production and secretion. However, this approach is associated with its challenges, as the simultaneous expression of several enzymes at optimal levels is required for the hydrolysis of cellulose. According to the CAZyme database, at least fifteen families of glycosyl hydrolases (GHs), can degrade cellulose [24]. Filamentous fungi have a complex regulatory system to control the expression of these cellulase encoding genes as well as genes that participate in the sensing and intracellular transport of monomeric sugars present in the medium (Fig. 1). An extensive analysis of the GH genes and their promoter regions has enabled the identification of several transcription factors that are key components in this regulatory network [25]. As these transcription factors interact with multiple genes, any modification to their sequence or expression is expected to have widespread outcomes; this review, therefore, focuses on the molecular mechanism of action of these transcription factors and their effect on the expression of cellulases and hemicellulases.

## Genomes and Carbohydrate-active enzymes (CAZymes) of cellulase producing fungi

Several strains belonging to the genus *Trichoderma* have been extensively investigated for their ability to produce cellulases, and their genome sequence data has been widely used by researchers across the world for carrying out further strain improvements via genetic engineering. The *Trichoderma reesei* QM6a was one of the first fungal strains to be isolated, and whose mutagenesis gave rise to important commercial variants such as the RutC30 strain. A comparative evaluation of the genome sequences of the two strains (QM6a and RutC30) revealed that the genome size in the mutant was smaller than the parent by ~ 1.4 Mb due to a truncation in the catabolic repressor protein—Cre1, a frame-shift in the beta-glucosidase II gene, and the loss of an 85 kb region containing 29 genes mainly associated with the primary metabolic pathways [26]. On the contrary, a comparison of the cellulolytic *T. reesei* QM6a genome with the genomes of mycoparasitic strains of *Trichoderma* (such as *T. atroviride* and *T. virens*) indicated that the latter had larger genome sizes, probably due to differences in the environmental conditions under which these organisms naturally thrive [27, 28]. In addition, it was also observed that several DNA repair-related genes had been lost during the evolutionary process in *T. reesei* but which were retained in both *T. atroviride* and *T. virens* [29]. One of the prominently affected DNA repair pathways is the one that repairs alkylated DNA, with *T. reesei* having only one ada gene (ada1) while *T. virens* and *T. atroviride* had two and four copies, respectively. Similarly, *T. reesei* had only one Mgt1/Ogt ortholog, while both *T. atroviride* and *T. virens* had two copies [29]. The impaired DNA repair system in *T. reesei* could have made it more susceptible to both chemical/physical mutagenesis and targeted strain improvement efforts, thus making it the preferred strain for genetic engineering as compared to other *Trichoderma* strains.

**Fig. 2:**
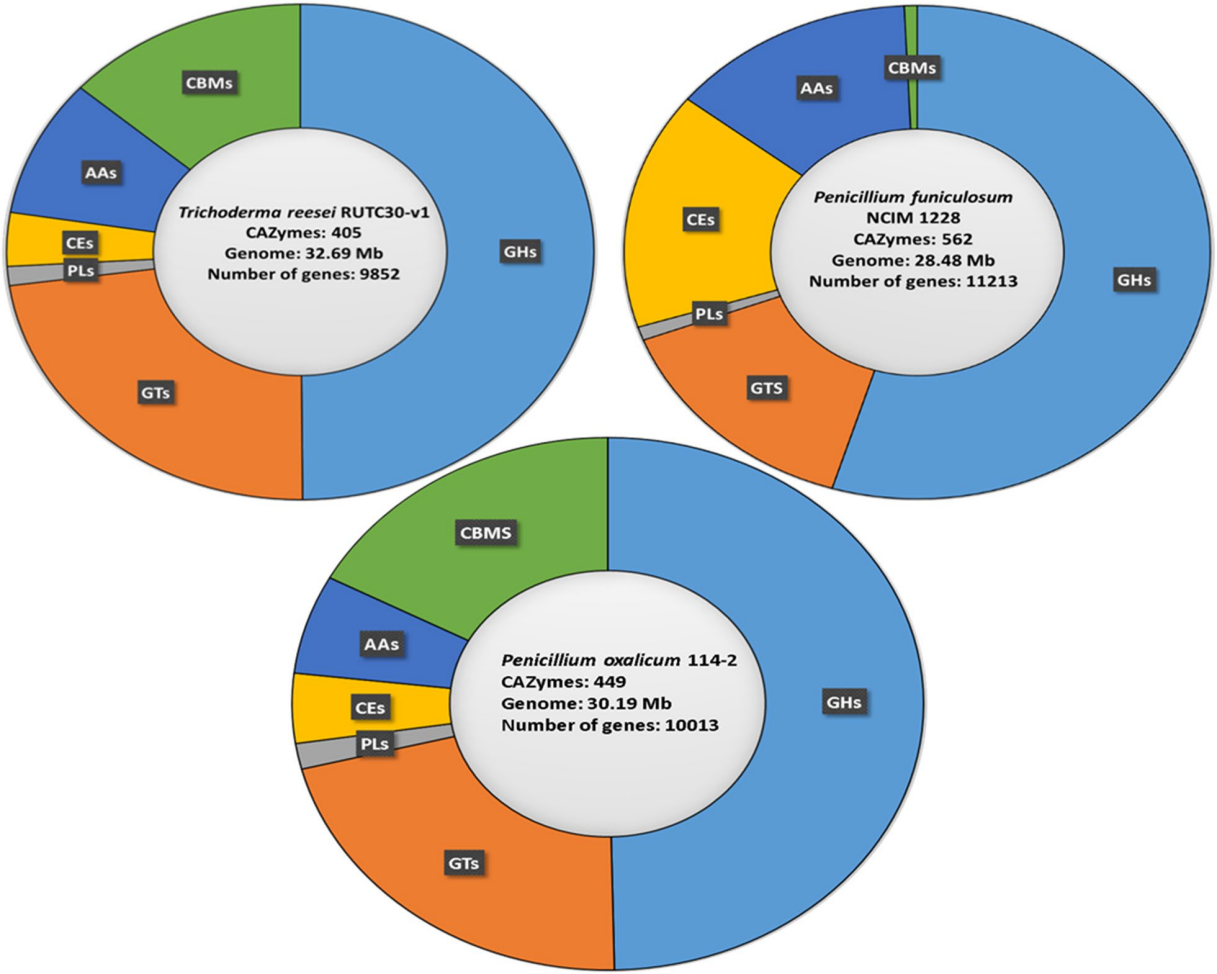
Comparative analysis of genome with respect to CAZymes in *Trichoderma reesei*, *Penicillium funiculosum* and *Penicillium oxalicum* 114–2. *GHs* Glycoside hydrolases, *GTs* Glycosyl transferase, *PLs* Polysaccharide Lyases, *CEs* carbohydrate esterases, *AAS* auxiliary activities, and *CBMs* carbohydrate binding modules

The genome data has also been used to identify key genes such as transcriptional factors and transporters that control the induction and expression of carbohydrate-active enzymes (CAZymes) and plant cell wall degradation enzymes [28–31]. *T. reesei* RutC30 has around 200 GHs and 355 CAZymes that include several types of enzymes such as glycosyl transferases (GT), polysaccharide lyases (PL), carbohydrate esterases (CE), auxiliary activities (AA), and carbohydrate binding modules (CBM), etc. (Fig. 2) [32].

Another filamentous fungus that has been widely studied for cellulase production is *Penicillium funiculosum* (or *Talaromyces funiculosus*), with a genome size of 28.48 Mb, having 21 scaffolds and 11,213 predicted genes [https://www.ncbi.nlm.nih.gov/genome/76218? genome_assembly_id = 454613]. The *P. funiculosum* genome contains around 92 GHs and 113 CAZYmes (Fig. 1) [33], which is much lower than the number of CAZymes in *T. reesei*. Both *P. funiculosum* and *T. reesei* demonstrate high cellulase production capacity even though the number of CAZyme coding genes is substantially different, which indicates the importance of the regulatory network in protein secretion in filamentous fungi, and the need to have a deeper understanding of the various transcription factors involved. The following sections focus on the transcription factors participating in the regulation of expression of cellulase genes, and their molecular mechanism of action.

## Transcriptional regulators involved in the regulation of cellulolytic and xylanolytic gene expression in filamentous fungi

Over the past two decades, significant progress has been made in elucidating the mechanisms involved in the transcriptional regulation of cellulolytic and xylanolytic genes in filamentous fungi. Analysis of the expression profiles of cellulase and xylanase genes as well as their promoter regions have led to the identification of several cis- and trans-acting elements that control cellulase gene expression. The major transcription factors participating in the regulation of the cellulase and xylanase gene expression and their functions are discussed in the following sections and also summarized in Table 1, while the genetic engineering strategies involving the modification of transcription factors have been compiled in Table 2.

**Table 1:** Transcription factors involved in cellulase expression and their binding sites

Transcription factor	Motif	Consensus sequence	Function	References
Xyr1	Zn_2_Cys_6_	GGCTRRR or GGC(A/T)3	(Hemi)-cellulose utilization	[34, 39]
Ace2	Zn_2_Cys_6_	GGCTAATAA or GGC(T/A)4 or XAE	Cellulose utilization	[38]
Ace3	Zn_2_Cys_6_	5′GGCTAA-3′	Cellulose utilization	[61]
BglR	Zn_2_Cys_6_	unknown	Sugar sensing and betaglucosidase repressor	[65]
Hap2/3/5	CCAAT-binding factor (CBF)	CCAAT	Chromatin remodeling, respiratory metabolism and CAZy regulation	[76, 78]
CreA/Cre1	Cys_2_His_2_	SYGGRG	Carbon catabolite repression	[87]
Ace1	Cys_2_His_2_	5′ AGGCA-3′	Cellulase repression	[103, 104]
Xpp1	E-box–HLH	WCTAGW + AGAA	Primary, secondary metabolism switch and xylanase represssor	[111, 112]
Vel1	Velvet	Unknown	Light response, secondary metabolism response, (a)sexual development	[121, 122, 124]
Pac1	Cys2His2	GCCARG	Alkaline pH response	[132, 133]
Clr1, Clr2	Cys_2_His_2_	Unknown	Light dependent xylan and pectin utilization	[136–138]
Rxe1	Cys_2_His_2_	Unknown	Cellulose utilization	[144]
Are1	Unknown	GATA	Protease and cellulase production	[145]
Ctf1	Unknown	5′‐GTTGYAHGAGGG‐3′	Cellulase repressor	[146]
Crz1	Unknown	5′‐[T/G]GGCG‐3′ or 5′‐GGGC [G/T]‐3′	Ca2 + /calmodulin‐calcineurin‐mediated cellulase utilization	[148]
Azf1	Cys2His2	5′‐AAGAAGACAGAG‐3′, 5′‐AAGAAGAA‐3′, 5′‐AACACGGAGGAG‐3′	Cellulose utilization	[149]
Vib1	NDT80-like DNA binding domain	Unknown	Cellulose utilization	[139, 140]

**Table 2:** Strategies for engineering cellulase expression in *Trichoderma reesei* and *Penicillium* sp

Transcription factor	Organism	Strategy	Observation	References
Xyr1	*T. reesei*	Overexpression of *xyr1* under the control of tcu1 promoter	Cellulase activities in presence of glucose and glycerol; no catabolite repression	[44]
	*T. reesei*	Overexpression of *xyr1*	Cellulase expression in presence of D-xylose; no catabolite repression	[45]
	*T. reesei*	Overexpression of xyr1 under the control of constitutive *pdc* promoter along with downregulation of Ace1	Significant enhancement in cellulase activity	[46]
	*T. reesei*	Expression of fused transcription factor with DNA-binding domain of *xyr1* and the transactivation domain of *ypr1* or *ypr2*	Expression of xylanases and cellulases in the presence of non-inducing carbon sources	[48]
	*T. reesei*	Deletion of *xyr1*	Reduction of cellobiohydrolase, β-xylosidase, β-glucosidase, α-arabinofuranosidase, α-galactosidase and β-galactosidase activities	[43]
	*T. harzianum*	Constitutive expression of xyr1 under *pkiI* promoter	threefold increase in FPase and 1.5-fold increase in β-glucosidase activities, which resulted in 26-fold improvement in saccharification	[55]
Xyr1	*P. funiculosum*	Deletion (*ΔxlnR)* and overexpression (*xlnR* +) of the *xlnR* gene	Reduced mycelial growth in *ΔxlnR* strain Increased protein secretion and expression of xylanases and endoglucanses in *xlnR* + strain	[56]
XlnR	*P. oxalicum*	Overexpression of mutated *xlnR* (A871V) with ClrB and deletion of *creA* Expression of cellulase genes *cbh1, cbh2* and *eg1*	8.9-fold increased cellulase expression and 51.5-fold increase in xylanase production	[57]
XlnR	*P. oxalicum*	Overexpression of *T. reesei* (x*yr1*) and *Neurospora crassa* (*xlr-1*), with their active mutants (*xyr1*^*A824V*^ and *xlr-1*^*A828V*^)	Increased cellulase production by 2.8 fold	[58]
Ace2	*T. reesei*	Deletion of *ace2*	Reduction in cellobiohydrolases I, II and endoglucanase activities by 30–70%	[59]
	*T. reesei*	Disruption of *ace2*	Reduction of cellulase expression in presence of cellulose, but not in the presence of α-sophorose	[25]
	*T. reesei*	Overexpression of Ace2	twofold increase in cellulase activities	[60]
Ace3	*T. reesei*	Overexpression of Ace3 Deletion of Ace3	1.5-fold increase in cellulase and xylanase activity Reduction in cellulase and hemicellulase activities and *xyr1* expression	[31]
	*T. reesei*	Deletion of Ace3	Reduction in cellulase and hemicellulase production, Downregulation of *xyr1* and 22 cellulase-related and 15 hemicellulase-related genes	[61]
	*T. orientalis*	Overexpression of *ace3* and *xyr1*	2.34-fold improvement in cellulase secretion twofold increase in FPase and CMCase with glucose as carbon source	[64]
Cre1	*T. reesei*	*cre1* binding sites of cbh1 promoter replaced with ace2 and Hap2/3/5 complex	Increased FPase and CMCase activities 39% and 30%, respectiverly	[95]
	*T. reesei, A. nidulans*	Deletion or truncation of cre1	Smaller colonies with fewer aerial hyphae and spores; cellulase and hemicellulase production in presence of glucose	[94]
	*T. reesei*	Deletion of cre1 and truncation (cre 1–96)	Morphological abnormalities leading to reduced growth twofold increase in cellulase activities	[96]
Mig1	*P. funiculosum NCIM1228*	Replacement of MiG1 with truncated Mig1^88^	twofold higher cellulase production in strain with truncated Mig1	[83]
Ace 1	*T. reesei*	Deletion of *ace1*	Increased expression of cbh1, cbh2, egl1, xyn1 and xyn2 inducing conditions (cellulose or sophorose)	[104]
Xpp1	*T. reesei*	Deletion of *xpp1*	Reduced expression of xyn1, xyn2, and bxl2	[112]
SxlR	*T. reesei*	Deletion of *sxlR*	1.4-fold increased in xylanase activity	[114]
Pac1	*T. reesei*	Deletion of *pac1*	fivefold increase in cbh and bgl expression	[133]
CLR1 and CLR2	*N. crassa*	Overexpression of *clr-2*	Expression and secretion cellulases in presence of glucose	[134]
Vib1	*T. reesei*	Overexpression of *vib-1*	200% increase in the cellulases secretion	[142]
Rxe1	*T. reesei*	Knockdown of rxe1 using a Cu-mediated RNAi system	Defective conidiation and reducd expression of Xyr1 and cellulase genes	[144]
Are1	*T. reesei*	Deletion of *are1*	Significant reduction in the expression and secretion of proteases	[145]
Ctf1	*T. reesei*	Deletion of the *ctf1*	36.9% increase in cellulase production	[146]
Rce2	*T. reesei*	Overexpression of Rce2	Reduced cellulase and hemicellulase production	[147]
Azf1	*T. reesei*	Deletion of *Azf1*	Reduced cellulase expression	[149]

### Xylanase regulator 1 (Xyr1)

The transcription factor Xyr1 was first identified as the major activator for both cellulase and xylanase genes and was isolated as an orthologue of *A. niger* XlnR [34]. The XyrI gene encodes a protein with 934 amino acids and a molecular mass of ~ 102 kDa. A detailed analysis of the genome sequences of *T. reesei* mutants led to the identification of the typical Zn2Cys6-binuclear cluster in this transcription factor [34, 35]. The deletion of Xyr1 has been shown to abolish the expression of the major cellulolytic (cbh1, cbh2, egl1, and bgl1) and xylanolytic (xyn1, xyn2, and bxl1) genes, irrespective of the inducer used (xylose, xylobiose, α-sophorose, and lactose) [36, 37], indicating its global effect on fungal metabolism.

Functional Xyr1 binding sequences are 5′-GGCTAA-3′ motifs arranged as an inverted repeat separated by a 10-bp spacer sequence within the xyn1 promoter region and an inverted repeat of a 5′-GGGTAA-3′ and a 5′-GGCTGG-3′ motif separated by a 12-bp spacer within the xyn2 promoter region, respectively. [34, 38]. Further analysis revealed that the target binding sequences of Xyr1 are the 5′-GGCWWW-3′ and 5′-GGCWWWW-3′ motifs [39]. The deletion of the Xyr1 gene has been shown to fully eliminate cellulase and xylanase production in *T. reesei*, whereas its constitutive expression significantly enhanced both cellulase and xylanase activities in the transformants [40, 41].

The detailed analysis of a cellulase negative mutant of *T. reesei* (QM9136) obtained through UV mutagenesis of QM6a [42] revealed a frame-shift mutation in the Xyr1 gene leading to the truncation of 140 amino acids at the C-terminal end. The re-transformation of QM9136 with the wild-type Xyr1 gene fully recovered its ability to produce cellulases [33]. In addition, the expression of the truncated Xyr1 gene i.e. without the C-terminal 140 amino acids in *T. reesei* QM9414 (a moderate cellulase producer) resulted in a cellulase-negative phenotype, indicating that this region containing the acidic activation domain is essential for the expression of both cellulases and hemicellulases, and which is, therefore, conserved across several *Trichoderma* sp. [33]. The deletion of Xyr1 in *T. reesei* led to a reduction in growth, as well as cellobiohydrolase and betaglucosidase activities, in addition to β-xylosidase and ɑ-arabinofuranosidase activities which were reduced by 97% and 47%, respectively in the recombinant strains [43].

Several studies have established the correlation between an elevated Xyr1 expression and an increase in cellulase and hemicellulase activities in *T. reesei*. For example, the overexpression of Xyr1 gene under the control of the tcu1 promoter resulted in the constitutive expression of cellulases; with the enzyme titers obtained using either glucose or glycerol as the carbon source, being similar to those achieved with Avicel using the parent strain [44]. Similarly, the overexpression of Xyr1 helped to partially overcome the repressive effects of D-xylose in *T. reesei* due to carbon catabolite repression (CCR) [45]. In another study, the constitutive expression of Xyr1 under the control of the strong *T. reesei* pdc promoter resulted in significantly enhanced cellulase activities [46]. The additional downregulation of the negative regulator, Ace1 further increased cellulase and xylanase activities, with the resulting strain exhibiting 103, 114, and 134% greater levels of total secreted protein, filter paper activity, and CMCase activity, respectively as compared to the parent *T. reesei* RutC30 strain [46]. The expression of Xyr1 could also be altered by the presence of lactose and/or galactose in the medium; and this effect was mediated by the truncated Cre1 gene in the *T. reesei* CL847 hyper-producer strain [47]. In a different strategy, a chimeric transcription factor was created by fusing the DNA binding domain of Xyr1 with the transactivation domain of ypr1 or ypr2 resulting in a highly trans-activating factor that significantly enhanced both cellulase and xylanase expression [48]. In a recent study, the overexpression of Xyr1 in a *T. reesei* strain lacking four major cellulase genes resulted in a completely different secretome profile in the presence of glucose or lactose, as compared to the wild type *T. reesei* strain. Increased expression of endoglucanases, betaglucosidases, arabinofuranosidases and other non-hydrolytic proteins as well was observed, all of which contributed to a greater hydrolytic potential of the crude enzyme even with untreated corn fiber [49].

The mechanism of the induction of cellulase expression by Xyr1 has been elucidated in a recent study where it was found that in the presence of cellulose, Xyr1 recruits TUP1, which forms a complex with CYC8 and binds to the promoter regions of target genes [50]. The CYC8-TUP1 complex has been previously reported to alter the chromatin structure by further recruiting chromatin modifying/remodeling complexes in *S. cerevisiae* [51–53]. In *T. reesei* too, it was found that the CYC8-TUP1 complex facilitated the binding of Xyr1 to the promoters of the target cellulase genes by chromatin remodeling (specifically, by mediating the loss of histone H4) [50]. The repression of either CYC8 or TUP1 led to complete loss of cellulase expression, in spite of Xyr1 overexpression which reconfirms the hypothesis that Xyr1 and CYC8-TUP1 regulate cellulase expression in an interdependent or synergistic manner, and are therefore crucial in ensuring the expression of cellulase-encoding genes over a sustained period [50]. In another recent study, it was shown that Xyr1 mediates the expression of target cellulase genes by binding to the promoters and recruiting Gal11 (a member of the Mediator complex), which further recruits RNA polymerase II to the site, leading to the expression of cellulase genes [54].

Although the role of Xyr1 as a global regulator of cellulase and hemicellulase expression in *T. reesei* is well studied, there are very few studies related to the effect of Xyr1 in other organisms. The constitutive expression of Xyr1 under the strong pkiI promoter in *T. harzianum* led to a threefold higher filter paper activity, as well as an increase in xylanase and betaglucosidase activities, all of which resulted in a significant improvement (~ 26 fold) in the saccharification of sugarcane bagasse as compared to the parent strain [55]. The role of the Xyr1 analog in *P. funiculosum* viz. XlnR was demonstrated by the use of two mutant strains—ΔxlnR and xlnR + (deletion and overexpression of the xlnR gene, respectively). The xlnR + strain showed higher mycelial growth and increased expression of xylanases and endoglucanases as compared to the ΔxlnR strain [56]. In another study, the overexpression of the XlnR gene with an A871V point mutation and ClrB, along with the deletion of creA resulted in a strain with 8.9- and 51.5-fold higher production of cellulases and xylanases, respectively [57]. The additional overexpression of cbh1, cbh2, and eg1 further enhanced cellulase production by ~ 13%. In another investigation, the overexpression of Xyr1 from *T. reesei* and XlrI from *Neurospora crassa*, as well as their constitutively active mutants (Xyr1A824V and Xlr1A828V) in *P. oxalicum* led to increased cellulase production as compared to the wild type strain [58]. Further, the expression of the mutant variants in *P. oxalicum* led to a 2.8 fold higher cellulase production as compared to the wild-type Xyr1 and XlrI genes. This could be attributed to the point mutation in the C-terminal activation domain reiterating its role in the effect of Xyr1 on the expression of cellulases and hemicellulases [58].

### Activator of cellulase expression (Ace 2)

The Ace2 gene was isolated from a cellulose-induced cDNA library of *T. reesei* using the yeast one-hybrid screening method [59]. Ace2 encodes a typical zinc binuclear cluster protein having 341 amino acids and its disruption led to reduced expression of all major cellulases and xylanases in the presence of cellulose, but not in presence of α-sophorose [25]. This suggests that although both cellulose and sophorose are potent inducers of cellulase expression, their mechanism of induction differs.

The DNA-binding domain of Ace2 binds to the 5′-GGCTAATAA-3′ sequences in the cbh1 promoter and 5 ′-GGGTAA-3 ′ sequences in the cbh2 and xyn2 promoter regions, during in vitro binding assays. In addition, the binding of the full-length Ace2 gene to an inverted repeat motif consisting of the 5′-GGGTAA-3′ and 5′-GGCTGG-3′ sequences in the xyn2 promoter region has also been demonstrated [38]. An Ace2 homolog has not yet been found in other fungal genomes as yet, suggesting that it could be specific to *Trichoderma* sp. The function of Ace2 was elucidated by knocking out the gene in the hyper-cellulolytic *T. reesei* ALKO2221 strain, which led to lowered expression of cbh1, cbh2, egl1, egl2, and xyn2 genes, and a 30–70% reduction in cellulase activities, even when grown in a medium containing Solka floc cellulose, which should ideally have induced the expression of cellulases [59]. However, cellulase induction by sophorose was not affected by the deletion of the Ace2 gene. The role of Ace2 as a positive regulator of cellulase expression was further confirmed in another study where the overexpression of Ace2 in *T. reesei* led to a twofold increase in cellulase activity in the recombinant T/Ace2-2 strain when grown in a medium containing a mixture of hardwoods supplemented with glucose or xylose. [60].

### Activator of cellulase expression 3 (Ace 3)

Ace3 is a typical Zn2Cys6 transcription factor isolated from *T. reesei* cultures induced by the addition of either Avicel or different lignocellulosic materials such as pretreated wheat straw, birch xylan, oat spelt xylan, differentially pretreated bagasse, etc. or specific disaccharides like sophorose [61]. The deletion of Ace3 led to a significant reduction in cellulase and hemicellulase activities, and lowered Xyr1 expression, suggesting that Ace3 is located upstream of Xyr1 in the induction mechanism of cellulase and hemicellulase [31]. Ace3 therefore, acts as a positive regulator of cellulases and xylanases, by regulating the transcription of Xyr1. It has recently been found that the expression of Ace3 is in turn modulated by the binding of the newly discovered Ace4 protein to the Ace3 promoter region [62]. In fact, the overexpression of Ace4 led to a 22% increase in the expression of the major cellulase genes, and this effect was mediated in an Ace3-dependent manner [62].

The deletion of Ace3 resulted in negligible expression of cbh1, cbh2, egl1, bgl1, and xyn3 in the recombinant strain as compared to the parental strain [31]. In another study, the deletion of Ace3 led to the downregulation of 22 cellulase-related and 15 hemicellulase-related genes; which included most major cellulase genes such as cbh1, cbh2, egl1, egl2, egl3, bgl2, etc. indicating the key role Ace3 plays in the regulation of cellulase and hemicellulose expression. [61]. The expression of a truncated Ace3 gene (having a deletion of 34 amino acids at the C-terminus) led to a complete loss of function, indicating that the active domain is located towards the C terminus [61]. This finding was further corroborated by a recent study in which different variants of Ace3, having a common Zn2Cys6 domain in the N-terminal and different truncations in the C-terminal region were expressed in *T. reesei*. It was observed that a truncation of 7–17 amino acids in the C-terminal region led to a 1.5–twofold increase in total cellulase production in presence of glucose, and a threefold increase in activity in presence of lactose, whereas a longer truncation of 20–25 amino acids led to severe reduction in protein production [63]. The authors have suggested two possibilities for this interesting observation—either the presence of an inhibitory domain in this region or the occurrence of a stretch of hydrophobic residues, which could interact with other transcription factors leading to reduced expression [63].

The constitutive overexpression of Ace3 and Xyr1 in *T. orientalis* EU7-22 strain resulted in a 2.34-, 0.68- and 1.06-fold increase in cellulase, xylanase, and protein secretion, respectively in the recombinant strain dxyA-8 as compared to the parent strain [64]. Moreover, the FPase and endoglucanase activities were also increased to 2.55 IU/mL and 90.38 IU/mL using glucose as the carbon source, which was 2.12 and 1.95 folds higher than those obtained in the presence of cellulose, suggesting that Ace3 could also have a role in overcoming CCR.

### Beta-glucosidase regulator (BglR)

BglR is a transcription factor that up-regulates the expression of specific genes encoding β-glucosidases [65]. A comparative genomic analysis to verify SNPs between the *T. reesei* mutant PC-3–7 and the parent KDG-12 strains confirmed the presence of BglR. A mutant lacking the BglR gene as well as the PC-3–7 mutant exhibited higher cellulase production during growth on cellobiose, while reversion of the missense mutation in BglR to the wild-type allele resulted in reduced cellulase production [65]. The mutant BglR strains showed reduced β-glucosidase activity even under inducing conditions, which indicated that BglR can up-regulate specific β-glucosidase genes (except for bgl1, which appears to be under the direct control of Xyr1) [65]. Although more insights into the function of BglR remain to be understood, it has been hypothesized that it plays a key role in the overproduction of cellulases especially when cellobiose is used as the sole carbon source [65]; this observation indicates that BglR could also be involved in glucose sensing.

### Hap complex

Hap complexes have been identified in *Aspergillus* sp. (HapB /C/E), *T. reesei* (Hap2/3/5), and *N. crassa* (Hap2/3/5) among other filamentous fungi [66, 67]. These complexes have been shown to modulate the expression of several genes such as the *A. nidulans* acetamidase gene (amdS), the *A. oryzae* Taka-amylase gene (taa), and the *T. reesei* cellulase and xylanase genes (*cbh2* and *xyn2*), etc. [68–72]. Their binding sequence viz*.* CCAAT is present in ~ 30% of all eukaryotic promoters and is usually observed 50–200 bp upstream of the transcription start site [73]. The first heteromeric protein complex bound to CCAAT sequences was found in *S. cerevisiae* [74, 75] and subsequently, similar CCAAT-binding complexes have been isolated and characterized in both fungi and plants. The Hap2/3/5 complex participates in the regulation of cellulase expression in *T. reesei* along with other regulators like Xyr1 and Ace2 [38, 76]. Interestingly, the Hap complex of *A. nidulans* helps in the formation of an open chromatin structure near the promoter region of the amdS gene [77]. Similarly, the binding of the Hap complex to the cbh2 activating element has also been reported to affect the nucleosome arrangement of the cbh2 promoter [78]. These findings indicate that the Hap complex affects cellulase expression indirectly by modifying the nucleosome structure, thus enabling the binding of other transcription factors to the promoter region of the target cellulase genes.

### Carbon catabolite repressor (Cre1)

Carbon catabolite repression (CCR) is a regulatory phenomenon observed in all living organisms wherein the expression of enzymes required for the assimilation of secondary carbon sources (such as polymers like cellulose, disaccharides, etc.) is inhibited when the preferred carbon source (usually glucose) is readily available in the medium [79–82]. In fungi, CCR occurs mainly through the Mig1/CreA/CRE1/Cre1 Cys2–His2 double zinc finger transcription factor that is well conserved throughout the fungal kingdom [83, 84]. In *T. reesei*, Cre1 was isolated as an ortholog of the *A. nidulans* and *A. niger* CreA gene [85]. The phosphorylation of the Ser241 residue located in the conserved acidic region of Cre1 by casein kinase II-like protein in the presence of D-glucose is essential for its binding to DNA [86]. The consensus binding sequence for Cre1 has been determined as 5′-SYGGRG-3′ [87] and two closely spaced motifs have been identified as the functional binding sites in vivo.

Although Cre1 regulates the expression of cbh1 and xyn1 [87, 88], its binding sites have not yet been identified in the promoter regions of cbh2 and xyn2 [71, 78]. Complementation experiments in the *T. reesei* RutC30 strain in which the truncated Cre1 gene was replaced with the full-length gene, confirmed that the repression of cellulase and hemicellulase genes is mediated by Cre1 [89, 90]. PC-3–7, a hyper-cellulolytic *T. reesei* mutant with a single-nucleotide mutation in the DNA binding domain of Cre1, showed a partial release of the strain from CCR [91]. Cre2, an orthologue of the CreB gene of *A. nidulans*, is a ubiquitin C-terminal hydrolase that might be involved in the de-ubiquitination of Cre1 in *T. reesei* [92]. In *A. nidulans*, CreB is stabilized by forming a complex with CreC [93]. Since the ortholog of CreC exists in the *T. reesei* genome as well, it is believed that the mechanism of CCR could be conserved between these two species.

The deletion or truncation of Cre1 resulted in altered morphology with smaller colonies having fewer aerial hyphae and spores as compared with the parental strains, as well as cellulase and hemicellulase production in the presence of glucose i.e. under inhibitory conditions [94]. The crucial role of Cre1 in regulating cellulase expression was further demonstrated by replacing its binding sites in the cbh1 promoter, with the reported binding sites of Ace2 and the Hap2/3/5 complex, which resulted in a 5.5-fold increase in the expression of the green fluorescence protein (GFP) reporter gene under inducing conditions i.e. in the presence of a mixture of cellulose and wheat bran, and a 7.4-fold increase in GFP expression under repressing conditions i.e. when glucose was used [95]. The cellobiohydrolase and endoglucanase activities also increased by 39% and 30% in the transformant strain. In another study, the deletion of Cre1 in *T. reesei* led to significant growth impairment in the transformant; however, the expression of a truncated version of the Cre1 gene resulted in enhanced cellulase production without any effect on growth [96]. It is understood that the CCR effect is mediated by protein kinases that phosphorylate a series of serine and threonine residues in the C-terminal region of Cre1, in response to the presence of glucose in the medium [86]. As the truncated Cre1 gene lacked this region, its expression led to cellulase production even in the presence of glucose. The key role of Cre1 phosphorylation in the CCR phenomenon was demonstrated recently in another study, in which four Ser/Thr residues in the C-terminal region were mutated to valine, to mimic the dephosphorylated condition. The S388V mutation resulted in a 2.25-fold increase in filter paper activity as compared to the parent *T. reesei* strain in the presence of glucose, and a 3.5-fold increase in activity when a mixture of Avicel and glucose were used, suggesting that phosphorylation of the Ser388 residue is critical for repressing cellulase expression in the presence of glucose [97]. Thus, inhibiting the phosphorylation of the C-terminal region of Cre1 could be another promising strategy to increase cellulase production without affecting growth. Another approach for relieving catabolite repression involves the deletion of the alpha-tubulin tubB gene in *T. reesei*, which led to the upregulation of several cellulase and hemicellulase genes, as well as genes encoding transporters of cellobiose and other sugars, when grown in a medium containing either glucose or cellobiose, suggesting that tubB could be involved in sugar sensing as well, and therefore analogous to Cre1 [98].

The repression of cellulase expression in presence of glucose in *Aspergillus* sp. is mediated by the CreA gene which contains DNA-binding zinc finger domains in the N-terminal region and the regulatory domain in the C-terminal region [99–102]. MigI the homolog of CreA in *P. funiculosum* also has a similar structure in which the N-terminal zinc finger domains are separated from the regulatory domains by an alanine linker. The expression of a truncated MigI^88^ gene with a disruption in the zinc finger domain resulted in increased hyphal growth and branching, and a 1.75-fold increase in glucose utilization. This was accompanied by a twofold increase in cellulase production and prolonged induction of cellulases in the presence of glucose, clearly suggesting that the mechanism of cellulase repression is more or less conserved across all filamentous fungi [83].

### Activator of cellulase expression 1 (Ace 1)

Ace1 was isolated from a cellulose-induced cDNA library of *T. reesei* along with Ace2 using the yeast one-hybrid screening method, based on its ability to activate the transcription of the *T. reesei* cbh1 promoter fused to the *S. cerevisiae* reporter gene his3 [103]. Ace1 is a class I zinc finger protein containing three Cys2His2-type zinc finger domains that bind to 5′-AGGCA-3′ motifs within the cbh1 promoter region and a 5′-GGCTAA-3′ motif within the xyn1 promoter region [34, 103, 104]. Although Ace1 activated the cbh1 promoter in *S. cerevisiae* during the yeast one-hybrid screening assay, its deletion from the *T. reesei* genome resulted in the increased expression of all major cellulase (cbh1, cbh2, and egl1) and xylanase genes (xyn1 and xyn2) when either α-sophorose or cellulose was used as the carbon source [104]. This discrepancy between the effects of Ace1 observed in *S. cerevisiae* and *T. reesei* has been attributed to the fact that the Ace1 expressed from the yeast expression library was truncated and lacked 242 amino acids from the N-terminus, which suggests that the N terminal region of Ace1 could be responsible for the repression of cellulases [104]. However, the deletion of Ace1 in a *T. reesei* strain overexpressing Xyr1 did not result in higher cellulase production as expected [105]. The authors have attributed this unusual observation to the reduced interaction between Xyr1 and Ace1, as it has been previously reported that these two transcription factors can form a complex and bind to the GGCTAA motif present in the promoter regions of target genes [34].

Ace1 acts as a repressor for cellulase and xylanase production in both *T. reesei* and *T. koningii* [40, 104, 106]. In contrast, its homolog in *Talaromyces cellulolyticus* viz. tacA induces the expression of cellulases, xylanases, and cutinases [107]. However, the TacA protein shows a low similarity to Ace1 except for the zinc finger domain, suggesting that it might be a novel transcriptional regulator [107]. Orthologs of Ace1 have been identified in several fungi [108]. Among them, the *A. nidulans* stzA gene which encodes an abiotic stress response regulator that reduces sensitivity to salt and DNA-damaging agents has been well characterized [109, 110]. Ace1 appears to have a more general regulatory role in addition to the repression of cellulases and xylanases. Although AceI is conserved across several cellulolytic fungi, it has not been fully characterized in other species as yet, and more insights regarding its mechanism are needed.

### Xpp1

The Xpp1 transcription factor was characterized in *T. reesei* due to its role in the repression of the xylanase-encoding genes (xyn1, xyn2, and bxl2) during growth on glucose or xylose, although no such effect was observed when cellulose was used as the carbon source [111]. Another study by the same group showed that Xpp1 acts as the switch between primary and secondary fungal metabolism, by promoting primary metabolism and fungal growth, while simultaneously repressing secondary metabolism. Xpp1 is a broad-spectrum transcription factor that can regulate the expression of around 995 genes including 28 transcription factors, and the repression of xylanases appears to be only a secondary effect [112]. Xpp1 is a basic helix-loop-helix protein (bHLH) with an E-box domain that typically binds to the hexameric palindrome 5′-CANNTG-3′ [113]. Experimental observations suggest that the actual binding site is composed of the hexameric palindrome 5′-WCTAGW-3′ along with an inverted AGAA-repeat [111]. It has been observed that Xpp1 regulates the transcription of hemicellulase genes only in the later stages of growth and no significant difference in xylanase activities was observed in an xpp1-disrupted strain or its parent strain, in the initial 72 h, reconfirming its role as a repressor of secondary metabolism [112].

### SxlR

The SxlR transcription factor was identified due to its role in inhibiting the expression of the major xylanases such as xyn1, xyn2, xyn5, etc. while not affecting cellulase expression in *T. reesei* [114]. The SxlR deleted strain showed a 4.6-fold increase in the transcription levels of xyn2 after around 12 h of growth in xylan-containing medium [114]. Although Xpp1 and SxlR appear to have a similar role i.e. the repression of xylanases, their mechanism of action, as well as their consensus binding sites differ [111].

### Velvet complex

The Velvet complex is a heterotrimeric complex consisting of the VelA, VelB, and LaeA proteins, that controls secondary metabolism and the switch between asexual to sexual reproduction, in response to changes in illumination [115–121]. In *T. reesei* asexual development is preferred under dark conditions, and sexual growth under illuminated conditions [122], which is in direct contrast to *Aspergillus* sp. [123]. The *T. reesei* homologs viz. Ve1, Vel2, and Vel3 are involved in colony morphogenesis, hyphal polarity, and branching, and the deletion of either Ve1 or Vel2 led to the loss of pigmentation, reduced sporulation, and the formation of small, irregular colonies [124]. In addition, a significant downregulation of xyr1, cbh1, cbh2, egl1, egl2, and bgl genes was observed, which suggested that Ve1 and Vel2 regulate cellulase expression by binding to the Xyr1 promoter and inhibiting its expression [125].

### Pac1

The ambient pH of the growth medium is known to affect the growth and the types/amounts of secreted proteins in filamentous fungi. The pH signal transduction pathway has been extensively investigated in *A. nidulans*; it contains six pal proteins (PalA, B, C, F, H, and I) and the transcription factor PacC that activates the expression of alkali-responsive genes and represses the expression of acid-responsive genes under alkaline conditions [126–131]. Orthologs of PacC and the six pal proteins have also been found in *T. reesei* and several other filamentous fungi [132, 133]. A large number of genes in *T. reesei*, such as those encoding transporters, signaling-related proteins, extracellular enzymes, and proteins involved in different metabolic functions, as well as some cellulases and hemicellulases are pH-responsive [25]. For example, the xylanase genes—xyn2, xyn3, and GH30 are abundantly expressed at high pH, whereas xyn1 and xyn5 are expressed more at a lower pH [25]. The deletion of pac1 significantly increased the expression levels of the major cellulase genes and consequently, cellulase activities at neutral pH [133]. This suggests that Pac1 is indirectly involved in the regulation of cellulase production. In addition, the expression levels of Xyr1 and Ace2 also increased in the ∆pac1 mutant at neutral pH. Although a variety of glycoside hydrolase genes respond to changes in pH, only a few are under the regulation of Pac1. It has been hypothesized that other regulatory mechanisms have a stronger effect on cellulase and hemicellulase gene expression and hence, mask the effect of Pac1 regulation [133].

### Clr1 and Clr2

The transcription factors Clr-1 and Clr-2 were identified as they were essential for the growth of *T. reesei* on cellulose and could induce the expression of all major cellulase and hemicellulase genes [134]. Both Clr1 and Clr2 have a zinc binuclear cluster that participates in DNA binding, and a middle homology domain or the activation domain [134]. Clr1 controls the expression of Clr2, which then plays a critical role in regulating mannan degradation and cellulase expression, by binding to the promoter regions of the target genes as a heterocomplex with Clr1 [134–137]. Clr1 and Clr2 are critical components of the cellulose, cellobiose and glucose-sensing mechanisms of most ascomycetes and could play a role in initiating the CCR pathway [134]. The presence of Clr1 in *T. reesei* was confirmed in a recent study, where it was observed that it had very low similarity to other ascomycetes, indicating its possible recent evolution [138]. In *T. reesei*, Clr1 and Clr2 also appear to have the additional role of light sensing, and the regulation of xylanase expression in response to varying light intensities. The deletion of Clr1 and Clr2 in *T. reesei* led to reduced Xyr1 and Xpp1 expression resulting in lowered cellulase and hemicellulase expression, suggesting that both Xyr1 and Xpp1 act downstream of Clr1-Clr2 [138].

### Vib1

Vib1 is a homolog of the *S. cerevisiae* NDT80 gene which acts as a transcriptional activator of genes involved in meiosis [139]. Vib1 appears to have multiple roles in the filamentous fungus *N. crassa*, where it led to the increased expression of extracellular proteases in response to carbon and nitrogen starvation [140]. It also controls cellulase production indirectly, by regulating the glucose sensing and CCR pathways, and by modulating the expression of regulators like Clr2 [141]. The overexpression of Vib1 in *T. reesei* RutC30 led to reduced conidiation but increased the secretion of both cellulases and total protein by 200% and 219%, respectively, when the strain was grown in both pure cellulose, and soluble inducers like lactose [142]. This significant improvement in enzyme production could be attributed to the global effects of Vib1, as the recombinant strain showed increased expression of all positive regulators viz. Xyr1, Ace2, Ace3 and reduced expression of the major negative regulators i.e. Cre1 and Ace1 [142]. On the other hand, the deletion of Vib1 led to decreased expression of 586 genes and increased expression of 431 genes, in the presence of cellulose [143]. Moreover, the transcriptome of the ΔVib1 strain was highly similar to that of ΔXyr1 strain, suggesting that Vib1 might be regulating cellulase expression in association with Xyr1 [143].

## Recent developments in identification of other transcription factors

In addition to the regulatory factors mentioned above, several transcription factors that can affect cellulase or hemicellulase expression indirectly, have been identified in the last few years using techniques like yeast hybrid screens, DNA binding assays, etc. Their exact mechanism of action remains to be elucidated; some have been found to have binding sites in the promoter regions of cellulases themselves, while others could interact with regulatory genes like Xyr1. A few studies related to these newly identified regulatory factors are listed below.

A yeast one-hybrid screen conducted to identify transcription factors binding to the Xyr1 promoter led to the discovery of the Rxe1 promoter [144]. Rxe1 homologs exist in several filamentous fungi but none of them have been assessed for their role in plant cell wall degradation. The knockdown of Rxe1 in *T. reesei* using a copper-mediated RNAi system caused a defect in conidiation as well as a reduction in the expression of Xyr1 and several major cellulase genes. The mutant strain was fully rescued by the constitutive expression of Xyr1 in terms of its cellulase expression, however, the defect in conidiation persisted [144].

The GATA transcription factor Are1, an orthologue of the *Aspergillus* global nitrogen regulator AreA in *T. reesei* was recently found to be involved in the regulation of both proteases and cellulases. Deletion of the Are1 gene led to complete repression of protease secretion, and quantitative RT-PCR analysis revealed a significant reduction in the expression of the protease genes Apw1 and Apw2 in the Δare1 strain, even when grown in a medium containing peptone as the nitrogen source [145]. In addition, the deletion of Are1 resulted in decreased cellulase production in the presence of inorganic nitrogen sources like ammonium sulphate [145].

Ctf1 is a novel repressor of cellulase expression in *T. reesei* that was identified through artificial zinc finger engineering [146]. The *T. reesei* RutC30 strain was transformed with an artificial zinc finger protein library, to obtain the M2 transformant having 67.2% and 35.3% higher filter paper and endoglucanase activities, respectively as compared to the parent strain. A quantitative RT-PCR analysis of the M2 strain showed significant downregulation of the Ctf1 gene, which led to a 36.9% increase in cellulase production. As expected, the overexpression of the Ctf1 gene under the constitutive pdc1 promoter led to significant repression of cellulase genes [146].

In another study, Rce2, a protein recently identified by a pull-down and mass spectrometry analysis was found to have similar binding sites in the promoter regions of target genes as Ace3, and its overexpression led to reduced cellulase expression i.e. Rce2 acted antagonistic to Ace3 in *T. reesei* with regards to cellulase induction and led to repressed cellulase and hemicellulase expression [147].

The calcineurin-responsive zinc finger transcription factor 1 or Crz1 was identified in *T. reesei* by gene disruption [148]. Electrophoretic mobility shift assays (EMSAs) in combination with chromatin immunoprecipitation (ChIP) confirmed that Crz1 could bind directly to the upstream regions of Xyr1 and cbh1. A DNase I footprinting assay further identified the putative binding consensus site as 5’-[T/G]GGCG-3’ or 5’-GGGC[G/T]-3’. Crz1 regulates the expression of cellulase genes in response to extracellular calcium levels [148].

The comparative analysis of the genomes of *T. reesei, A. nidulans*, and *S. cerevisiae* led to the identification of a new transcription factor Azf1 [149]. The knockout of Azf1 in *T. reesei* led to reduced cellulase expression in the presence of both avicel and sugarcane bagasse. ChIP-quantitative PCR techniques indicated that Azf1 directly binds to the promoter regions of the cellulase genes cel7a, cel45a, and accessory genes like swollenin. All of these recent studies suggest that our understanding of the fungal cellulase expression system is limited, and several players in this complex pathway are yet to be identified.

## Engineering of transporters and effect on gene expression

Filamentous fungi can metabolize complex polysaccharides due to their capacity to secrete high concentrations of several hydrolytic enzymes, in addition to a large range of sugar transporters that can efficiently transport the monomeric sugars generated, into the cell. These transporters can sense changes in the environment and initiate signaling pathways, which in turn affect the expression of CAZymes. Transporters in filamentous fungi can be classified into the ATP-binding cassette (ABC) family and the Major facilitator superfamily (MFS), based on their mechanisms of action, and requirement of energy during membrane transport [150]. These families include almost half of the genes involved in transmembrane transport in fungi [150]. The MFS proteins are secondary transporters, whereas ABC proteins are primary active transporters, responsible for transporting a diverse range of substrates using the energy released during ATP hydrolysis [151].

The ABC superfamily contains 45 known sub-families, most of which are prokaryotic [151]. Sugar transport has not been associated with ABC transporters in eukaryotes, as of now [152]. In contrast, the *T. reesei* genome contains approximately 164 predicted MFS transporters [153, 154]. However, their involvement in sugar uptake has not been fully characterized as yet. MFS transporters can recognize and transport more than one type of sugar (such as xylose and cellobiose) into the cell [155]. For instance, *T. reesei* STP1 is involved in the uptake of both glucose and cellobiose [156]. Similarly, the *A. nidulans* transporter XtrD could transport several other monosaccharides, in addition to xylose and glucose [157]. These monomeric sugars once internalized, can trigger a metabolic signaling cascade that affects the induction/expression of cellulases [156]. Despite significant progress made in studies related to the production of cellulases, the influence of sugar transporters on the degradation of cellulose or lignocellulosic biomass has not been investigated in detail.

The deletion of the MFS protein Stp1 that transports cellobiose in *T. reesei*, resulted in the repression of both cellulase and hemicellulase genes, in presence of Avicel; however, no effect on cellulase expression was observed when the strain was grown in cellobiose. This indicates that multiple transporters could be involved in the uptake of cellobiose from the growth medium [156]. The Stp1 knockout strain had higher expression of two other MFS transporters. The deletion of one of these led to the identification of Crt1, which is required for growth (and cellulase activity) on cellulose or lactose but not required for growth (or hemicellulase activity) on xylan. The deletion of Crt1 did not affect the uptake of cellobiose or sophorose, indicating that its role in cellulase induction in *T. reesei* may involve other genes as well. This is further corroborated by the fact that phylogenetic analysis has revealed that orthologs of Crt1 exist in the genomes of many filamentous ascomycete fungi capable of degrading cellulose [156]. The Crt1 protein is now recognized as a high affinity transporter of cellobiose and lactose, similar to its analog in *N. crassa* Cdt1 and is believed to play a critical role in the cellulase induction signaling cascade. [158].

The sugar transporter Tr69957 capable of transporting xylose, mannose, and cellobiose in S*. cerevisiae* was identified in *T. reesei* by in silico analysis of RNASeq data. The deletion of this transporter in *T. reesei* affected fungal growth, biomass accumulation, and sugar uptake in the presence of mannose, cellobiose, and xylose. Further, the expression of cellobiohydrolases (cel7a and cel6a), β-glucosidases (cel3a and cel1a), and xylanases (xyn1 and xyn2) in the presence of both cellobiose and sugarcane bagasse were also adversely affected [159]. Another MFS family transporter TrSTR1, which was first identified in *S. cerevisiae* due to its role in xylose uptake, has now been found in *T. reesei*, where it has been associated with the utilization of xylose, arabinose, and their metabolites xylitol and arabitol [160]. The deletion of TrSTR1 led to the reduction of both xylanase activity, as well as total protein secretion; thus confirming the involvement of TrSTR1 in xylanase induction in *T. reesei* too [160].

Trhxt1, a putative glucose transporter gene was identified in *T. reesei* due to its repression at high glucose concentrations and expression at trace levels in the absence of glucose [161]. This gene could be induced during growth on cellulose when the glucose concentration generated during hydrolysis in the medium was is in the micromolar range. Further, this transporter could be down-regulated by either hypoxia or inhibition of electron flow in the respiratory chain by antimycin A. However, Trhxt1 could be strongly induced in the presence of high glucose concentrations under anoxic conditions, indicating that the mechanism of induction is much more complex than previously understood.

Several MFS family transporters are induced by lactose, and the deletion of fourteen such proteins led to the identification of a gene essential for both lactose uptake and utilization, as well as for cellulase induction by lactose in *T. reesei* [162]. In a similar study, two lactose permeases were identified to be associated with cellulase induction, in the industrial cellulase producing strain *T. reesei* PC-3–7 [163]. The deletion of these transporters led to decreased lactose uptake, leading to delayed growth and lower cellulase production when grown in lactose-containing media. However, no effect on growth or enzyme production was observed when these strains were grown in cellulose.

Cellodextrin transporters, namely, CdtC, CdtD, and CdtG, have also been associated with cellulase induction in *P. oxalicum* [164]. Although the deletion of a single cellodextrin transporter gene led to a slight decrease in cellobiose utilization, it had a negligible effect on cellulase expression, most probably due to the overlapping activity of the three isozymes. This was confirmed by the simultaneous deletion of cdtC and cdtD, which resulted in significantly reduced cellobiose consumption, poor growth on cellulose, and reduced expression of the major cellulase genes. In general, sugar transporters in filamentous fungi not only participate in the sensing and uptake of monomeric sugars or oligosaccharides from the environment, but also trigger a signaling cascade that leads to the induction of cellulases and hemicellulases. Understanding their mechanism of action therefore becomes critical for making sense of the complex phenomenon of cellulase induction and regulation.

## Conclusion

The on-site production of cellulolytic enzymes seems to be the most feasible solution to overcome the challenge of using expensive commercial cellulases for the hydrolysis of biomass, in 2G ethanol biorefineries. However, filamentous fungi like *T. reesei, P. funiculosum, P. oxalicum*, etc. that can produce cellulases and hemicellulases have several inherent limitations in their enzyme production and secretion capacity. These can be overcome by genetic engineering to develop strains that produce enzymes at an industrially relevant scale. Most of the research efforts in this direction have focused on the overexpression of key cellulase/hemicellulase genes. However, as we now understand, the regulatory network involved in the expression of cellulolytic genes in filamentous fungi is far more complex. With the knowledge available till date, it appears that the most important regulatory factors that promote cellulase expression are Xyr1, Ace2, Ace3 while the major inhibitory factors include Cre1 and Ace1, as their binding sites are observed in the promoter regions of the major cellulase and hemicellulase genes. Accordingly most of the research has also focused on the modification of these transcription factors. Other regulatory factors and transporters appear to have an accessory role as their effect may be complemented or antagonized by other proteins in the pathway. This review is, therefore, an attempt to understand the intricate network of transcription factors, regulators, transporters, etc. that participate in this process, thereby providing more targets for the rational engineering of filamentous fungi to develop better strains, for the production of cellulases and hemicellulases.

## Data Availability

Not applicable.
